# Integrating a self-directed ultrasound curriculum for the internal medicine clerkship

**DOI:** 10.1186/s13089-024-00367-4

**Published:** 2024-03-05

**Authors:** Emily Signor, John Gerstenberger, Jennifer Cotton, Jorie Colbert-Getz, Katie Lappé

**Affiliations:** 1 Department of Internal Medicine, University of Utah School of Medicine, 30 N Mario Cappechi Dr., Salt Lake City, United States; 2 Department of Emergency Medicine, University of Utah School of Medicine, Salt Lake City, UT USA

**Keywords:** Point-of-care ultrasound, Clerkship, Curriculum, Undergraduate medical education, Internal medicine

## Abstract

**Background:**

Incorporating ultrasound into the clinical curriculum of undergraduate medical education has been limited by a need for faculty support. Without integration into the clinical learning environment, ultrasound skills become a stand-alone skill and may decline by the time of matriculation into residency. A less time intensive ultrasound curriculum is needed to preserve skills acquired in preclinical years. We aimed to create a self-directed ultrasound curriculum to support and assess students’ ability to acquire ultrasound images and to utilize ultrasound to inform clinical decision-making.

**Methods:**

Third year students completed the self-directed ultrasound curriculum during their required internal medicine clerkship. Students used Butterfly iQ+ portable ultrasound probes. The curriculum included online modules that focused on clinical application of ultrasound as well as image acquisition technique. Students were graded on image acquisition quality and setting, a patient write-up focused on clinical decision-making, and a multiple-choice quiz. Student feedback was gathered with an end-of-course survey. Faculty time was tracked.

**Results:**

One hundred and ten students participated. Students averaged 1.79 (scale 0–2; SD = 0.21) on image acquisition, 78% (SD = 15%) on the quiz, and all students passed the patient write-up. Most reported the curriculum improved their clinical reasoning (72%), learning of pathophysiology (69%), and patient care (55%). Faculty time to create the curriculum was approximately 45 h. Faculty time to grade student assignments was 38.5 h per year.

**Conclusions:**

Students were able to demonstrate adequate image acquisition, use of ultrasound to aid in clinical decision-making, and interpretation of ultrasound pathology with no in-person faculty instruction. Additionally, students reported improved learning of pathophysiology, clinical reasoning, and rapport with patients. The self-directed curriculum required less faculty time than prior descriptions of ultrasound curricula in the clinical years and could be considered at institutions that have limited faculty support.

**Supplementary Information:**

The online version contains supplementary material available at 10.1186/s13089-024-00367-4.

## Background

Point-of-care ultrasound (POCUS) improves speed of diagnosis, reduces costly, unnecessary, and potentially harmful imaging, and aids in procedures [1]. Given its increasingly recognized use across many clinical specialties, 79% of medical schools feel that incorporating ultrasound curriculum is important [2]. Ultrasound curricula are commonplace in the preclinical years [3], where students learn basic ultrasound skills that improve their understanding of anatomy and physiology [2]. However, attempts to incorporate ultrasound into the clinical curriculum of undergraduate medical education (UME), where essential diagnostic and clinical reasoning skills are acquired, has been hindered by the need for significant faculty support [3]. This likely contributes to the sharp decline in ultrasound use in clinical years [4]. 

Medical schools have tried different approaches to integrate ultrasound into the clinical years [5–8]. Most curricula depend on in-person didactic instruction with significant faculty development and support [9]. For example, one assessment of the resources needed for a successful fourth-year elective found nine ultrasound trained faculty were required to commit 25 h per student per month [5]. Another needs assessment found that 24 qualified instructors would be needed to implement a faculty-led, hands-on program in the first-year curriculum for a class of 184 students [10]. Nationally, a lack of ultrasound-trained faculty is cited among the top reasons for lack of incorporation of ultrasound curricula [3]. 

Given the resource limitations of many medical schools, a need exists to design an ultrasound curriculum in clinical courses that does not rely on significant faculty support. Without integration into the clinical learning environment, ultrasound becomes a stand-alone skill and may decline by matriculation into residency. To address this need, we piloted a self-directed ultrasound curriculum in the third-year internal medicine (IM) clerkship designed to support and assess students’ ability to acquire ultrasound images on patients and to use bedside ultrasound in clinical decision-making in patients presenting with cardiopulmonary complaints.

## Methods

### Setting and participants

Participants were third-year Spencer Fox Eccles School of Medicine – University of Utah (Utah-Eccles SOM) students who matriculated in fall 2019 and completed their required IM clerkship during academic year 2021–2022. Students checked out Butterfly iQ+ portable ultrasound probes with iPad mobile devices in groups of two during their IM clerkship. Later, additional grant funding resulted in each student receiving their own probe. During the IM clerkship, students spent four weeks on general inpatient wards, two weeks on inpatient subspecialty wards (Cardiology, Hematology, Oncology, or Pulmonology), and two weeks in a general medicine clinic.

### Design

#### Pre-clinical curriculum

Prior to starting their IM clerkship, students completed two years of preclinical ultrasound curriculum. The preclinical ultrasound curriculum included ten lectures and twenty-one hours of hands-on lab time. Lab time consisted of student small groups that completed a image acquisition lab guide by scanning themselves or a partner with a faculty or senior student teaching assistant. By the end of the second year, students were expected to independently acquire specific standard views on themselves or a student colleague, recognize the structures in these views, and identify focused pathology. These skills were assessed on Objective Structure Clinical Exams (OSCE) and quizzes throughout years 1–2. All student modeling for scanning was voluntary and free of coercion.

#### Self-directed ultrasound IM curriculum

The self-directed ultrasound curriculum for the IM clerkship included online modules on clinical applications of ultrasound, including: indications to perform a scan, troubleshooting difficult image acquisition, interpretation of images for specific pathologies, and integration of findings into medical decision-making. The modules were cardiopulmonary focused, which included pericardial effusions, systolic left ventricle failure, pleural effusions, volume assessment, pulmonary edema, and pneumonia.

##### Ultrasound image acquisition assignment

Students were required to record and submit clips to the Butterfly Network Cloud of the following views: cardiac parasternal long axis, cardiac subxiphoid, the pleura, costophrenic angles, and inferior vena cava. The ultrasound curriculum director for Utah-Eccles SOM graded all image submissions on a 0–2-point scale for image quality (e.g., image included all essential elements) and setting (e.g., depth, gain) (Table 1). Students had to achieve a minimum score of 1 in both categories to pass the assignment. Students who received a grade of 0 for either category were given narrative feedback on how to correct their image and were required to resubmit the image. Students were encouraged to reach out to faculty for bedside instruction during office hours if they had difficulty with image acquisition.

**Table 1:** Ultrasound image acquisition grading rubric

Points	Image Quality Grading Definition
0	Unable to acquire an image of the item
1	Acquired the item, but with significant room for improvement
2	Acquired the item with image containing all essential elements
Points	Image Setting Grading Definition
0	Acquired the item with inappropriate depth, gain, and setting
1	Acquired the item with at least one correct setting, gain, or depth
2	Acquired the item with appropriate setting, gain, and depth

##### Ultrasound patient write-up assignment

Students were responsible for selecting and performing the appropriate views to evaluate a patient with a chief complaint of chest pain or shortness of breath. The student completed a patient write-up with a focus on integrating ultrasound into patient care and medical decision-making. The assignment included the indication for performing the scan, interpretation of images acquired, development of a differential diagnosis, and description of how images influenced the differential diagnosis and management plan. The assignment was pass/fail and students received formative narrative feedback.

##### Quiz

As part of a clerkship data interpretation quiz, students completed a 10-item multiple choice quiz (MCQ) (Supplemental Material A). The quiz focused on image interpretation and medical decision-making based on their image interpretation.

### Program evaluation

To evaluate the self-directed ultrasound curriculum and inform future implementation, we determined the overall value by gathering student performance, student feedback, and faculty time.

Student performance was measured with the two graded assignments. We computed descriptive statistics for average patient assignment and quiz scores. The frequency of images needing remediation was also computed for the patient assignment. Faculty time to grade assessments and give feedback was tracked.

Student feedback was gathered with a required end-of-course survey (Supplemental Material B). Students in blocks 1–2 were asked to describe the positive impacts of the curriculum and any barriers to optimizing the experience. Free text responses were imported into Microsoft Excel for coding. Responses were coded using qualitative content analysis by two authors who reviewed all the qualitative data and created preliminary codebooks. The two authors then met to review discrepancies in codes and coding. The data was reviewed again by both to ensure that all data was well described by codes and that related codes were combined into themes. Steps were taken to ensure trustworthiness. Coding was conducted by multiple authors to explore multiple interpretations of the data and the authors engaged in peer debriefing. Code counts were calculated to describe the relative frequency of themes.

Students in blocks 3–6 were asked about the impact of the curriculum on a 4-point Likert scale ranging from 0 = not at all to 4 = extremetly (Supplemental Material B) and descriptive statistics were computed. The Likert scale survey questions were informed by free text responses provided by students in blocks 1 and 2.

Faculty time was tracked for grading the assignments. Total time and time per student were computed for the academic year (AY). This study was deemed exempt by the University of Utah Institutional Review Board.

## Results

There were 110 students who matriculated in fall 2019 and completed the IM clerkship in AY21-22. One student’s assignment was missing. Additionally, since course feedback was provided anonymously, we could not exclude ten students who completed the IM clerkship in AY21-22 but matriculated before fall 2019.

Students averaged 1.79 (SD = 0.21) on the image acquisition assignment. Table 2 provides mean ratings by quality and setting for each view. Twelve students remediated the assignment due to a score of 0, mostly due to costophrenic angle only (*n* = 8) or in combination with another view. All students were able to resubmit and pass the assignment using the narrative feedback provided without additional in-person instruction. Students averaged 78% (SD = 15%) on the quiz. All students received a pass on the patient write-up assignment.

**Table 2:** Mean ratings by quality and setting for each required view

	Quality		Image Setting	
View	*Mean*	*SD*	*Mean*	*SD*
Cardiac Parasternal Long Axis	1.89	0.37	1.90	0.30
Cardiac Subxiphoid	1.68	0.56	1.81	0.46
Anterior Lung Sliding	1.79	0.47	1.82	0.41
Costophrenic Angle	1.62	0.66	1.80	0.46
Inferior Vena Cava	1.90	0.36	1.73	0.51

Forty-one students completed the end-of-course survey in blocks 1–2. Seventy-seven students completed the end-of-course survey in blocks 3–6. Most students reported the curriculum moderately or extremely improved their clinical reasoning (72%), learning of pathophysiology (69%), student value on team (60%), and patient care (55%). Student free text responses regarding positive benefits of the ultrasound curriculum included “medical decision-making and patient care” and “opportunities for independent practice”. Primary barriers to learning were “equipment access and functionality” and “insufficient instruction and feedback” (Table 3).

**Table 3:** Themes identified in qualitative analysis of student comments

	No. of comments (%)	Representative quotation
**Positives of Curriculum**	45	
Medical decision-making and patient care	18 (40%)	“Having the US literally helped save someone’s life with a pericardial effusion who was moving toward tamponade.”
Opportunities for independent practice were valuable	8 (18%)	“Opportunities to practice with patients”
General ultrasound skill	7 (16%)	“Improved my ultrasound skills”
Understanding of anatomy and pathophysiology	5 (11%)	“We ultrasounded almost every patient and the pathophysiology I was supposed to be seeing made so much sense”
Student value on the medical team	4 (9%)	“It was an excellent way for me to shine during patient presentations, even table rounds when I was able to show the clips”
Assignments, online modules, and asynchronous feedback were valuable	3 (7%)	“The feedback I got from Dr. Cotton about my image submissions was EXTREMELY helpful”
**Barriers to Learning**	47	
Equipment access and functionality	10 (21%)	
*Gel Availability*	*5 (11%)*	“Wasn’t sure where to get ultrasound gel at first”
*Probe Availability*	*3 (6%)*	“We had to share probes which made it hard”
*Ipad/App Functionality*	*1 (2%)*	“Trouble with iPads and butterfly app”
*Image Quality*	*1 (2%)*	“The images I get on the nice ultrasound machines are nothing like the ones I get using the Butterfly”
Insufficient instruction and feedback	10 (27%)	“ I appreciate the effort that has been put into teaching us about ultrasounds, but it’s still just not nearly enough for me to be able to use them in a way that is useful to patient care”
Time	7 (19%)	“Finding time during the day to use it”
Faculty ultrasound knowledge and enthusiasm	6 (16%)	“Some attendings were excited about it, others didn’t really care that it was available”
Fear of harming or burdening patients	4 (11%)	“I found it difficult to explain to patients that I was going to ultrasound them exclusively for my own learning”

Faculty time for curriculum creation was approximately 45 h, which included module and assignment creation and meeting with course directors and administrators. Overall, the faculty time required for grading per student per clerkship block was 21 min and was performed at the faculty’s convenience. For a class of 110 students, this averaged to 38.5 h per year for grading and feedback.

## Discussion

For this IM clerkship self-directed ultrasound curriculum pilot, students were able to acquire ultrasound images of 5 cardiopulmonary views and explain their use of ultrasound to aid in the medical decision-making of a patient presenting with a cardiopulmonary complaint. Additionally, students demonstrated their ability to interpret cardiopulmonary ultrasound images via MCQ. End-of-course surveys highlighted students’ perceived benefits of the curriculum, including enhanced clinical reasoning and medical decision-making and improved understanding of anatomy and pathophysiology.

Our study has several strengths. Most notably, the self-directed ultrasound curriculum required minimal faculty time. Prior studies have relied heavily on in-person faculty instruction. Bahner, et al., described holding hands-on sessions twice per month for three hours under direct faculty supervision in addition to weekly intensive care unit teaching rounds supervised by faculty and monthly journal club [5]. For institutions with a preclinical ultrasound curriculum that lacks a repository of trained clinical faculty, our pilot provides a model of how to create a curriculum that can be easily scaled across large groups of students in the clinical environment. Despite little in-person faculty instruction, the majority of students independently obtained adequate images on their first attempt. For those whose initial images were inadequate on first pass, narrative feedback alone was sufficient for successful remediation.

Of note, 27% of students in our pilot desired more direct faculty instruction. This finding is not surprising, as ultrasound image acquisition can be more complex for patients in authentic clinical environments. The optimal amount of in-person faculty instruction is likely more than what we provided. To promote instruction in the clinical environment at our institution, 12 academic IM hospitalists received faculty development in POCUS in 2023. Additionally, the IM residency implemented POCUS curriculum for all first-year residents, starting in July 2023. By training residents and core faculty in POCUS, students will have improved access to in-person instruction and feedback in the clinical environment. With an increase in POCUS training amongst residency programs [11] as well as increased use of diagnostic and procedural POCUS in the inpatient environment amongst faculty, we expect that bedside instruction in POCUS will continue to improve in the future.

In addition to requiring less faculty time, our pilot required students to obtain images on hospitalized patients instead of standardized patients or other healthy individuals. This differs from many described clerkship-based curricula where most of the skills practice took place with live models or simulation [5, 6, 8, 12]. Our model tasked students with the real world limitations of image acquisition, such as maneuvering difficult windows, patient comfort, and time management skills, and promoted self-directed learning. Many of the barriers students noted, such as technology limitations and equipment availability, will continue to be faced by physicians using POCUS well into their careers.

Lastly, we found that students noted that the use of ultrasound improved their relationship with patients and helped them with medical decision-making and patient care. Although the patient perception of POCUS is not well studied, the perceived improvement in medical decision-making is consistent with prior data. One systematic review found that the use of POCUS resulted in the change of a main diagnosis or addition of a new diagnosis 18 and 24% of cases respectively and impacted management 37-52.1% of the time [13]. Our assessment of students’ medical decision-making did not look at patient outcomes; however, students were able to state the indication for ultrasound image acquisition, interpret the images, and describe how image acquisition affected the diagnosis and management of a patient with a cardiopulmonary chief complaint. While the curriculum described by Lum et al. found that students struggled with the use of ultrasound in medical decision-making on standardized patients, [12] their curriculum differed from this study in that our students were asked to practice skills on hospitalized patients with acute cardiopulmonary complaints.

Our study has several limitations. First, our pilot was single-centered; however, it was a requirement for all clerkship students, regardless of their interest in ultrasound. Second, there was significant cost. While grant funding allowed us to purchase Butterfly iQ+ probes for every student in the clerkship, different models of probe distribution could be considered. For example, one probe could be allocated per clinical site to be shared by students. Alternatively, the clerkship could purchase the number of probes needed for a single clerkship block and lend out probes to students. While having individual student probes likely contributed to the success of our pilot, there are more cost efficient means of probe distribution described above that would allow for implementation of the curriculum for programs with resource limitations. Third, a self-directed ultrasound curriculum requires students to have a preclinical ultrasound curriculum so that upon transition to the clinical curriculum, students have the skill to independently scan patients. Finally, images and assignments were graded by a single grader with a scoring system that is not validated, which could introduce bias. In future iterations of this curriculum, we could consider blind grading or grading of assignments by multiple graders.

## Conclusions

In conclusion, the self-directed ultrasound curriculum had overall value for student learning and was less time intense for faculty than prior descriptions of ultrasound curriculum in the clinical years. After completing self-directed modules, students were able to successfully obtain and interpret images on patients in the clinical environment. This curriculum could be considered at institutions that have limited faculty support for an ultrasound curriculum in clinical courses.

### Electronic supplementary material

Below is the link to the electronic supplementary material.

Supplementary Material 1

Supplementary Material 2

## Data Availability

The data that support the findings of this study are available from the corresponding author, KL, upon reasonable request.
