# Supplementary material for: Integrating a self-directed ultrasound curriculum for the internal medicine clerkship
Source: Ultrasound J. 2024 Mar 5;16:19. doi: 10.1186/s13089-024-00367-4 (PMC10914648; doi:10.1186/s13089-024-00367-4)
Supplement: Supplementary file 1 — Supplementary Material 1 [file 13089_2024_367_MOESM1_ESM.pdf]

Supplemental Material A: Multiple Choice Quiz

Note: Videos have been modified to representative screenshots

Correct answers are bolded below.

1. A 30 year old patient presents to your clinic. He has had one day of chest pain. He feels somewhat short of breath when he exerts himself. You note he is quite tall and has a pectus excavatum deformity when you examine his chest. His vitals signs are within normal limits except for a borderline oxygen saturation of 91% and a respiratory rate of 22. He does not appear in acute distress. You perform a cardiac exam to look for signs of a pericardial effusion, but there is none. You perform a lung exam and find the image below in his right anterior chest. What is the next step in management of this patient?

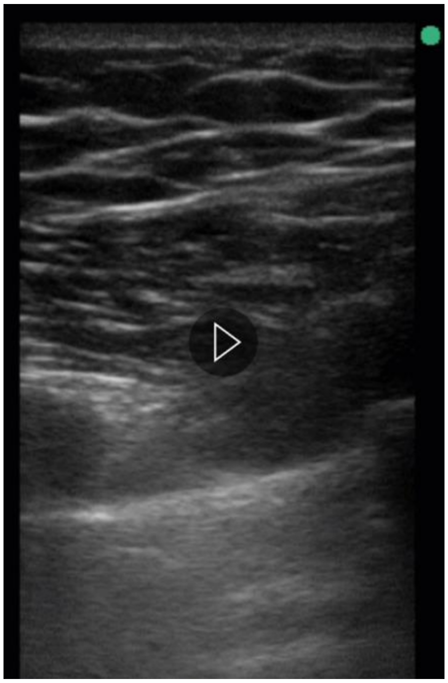

   a. Outpatient follow up with pulmonologist because of your positive point of care ultrasound findings
   b. Start patient on naproxen because a negative cardiac and lung ultrasound does not rule out pericarditis
   c. Send patient to emergency department for pigtail thoracostomy tube placement
   d. Order additional testing because of your negative point of care ultrasound findings

2. You are caring for an admitted patient who has developed gradually worsening shortness of breath. They have been admitted for hydration for inability to tolerate PO following their most recent chemotherapy for their lung cancer. Their vitals are normal except for a oxygen saturation of 88% with ambulation and mild tachypnea at rest. You call radiology who lets you know a chest xray will take a few hours to be performed. You find the following on ultrasound. What is causing this patient's shortness of breath?

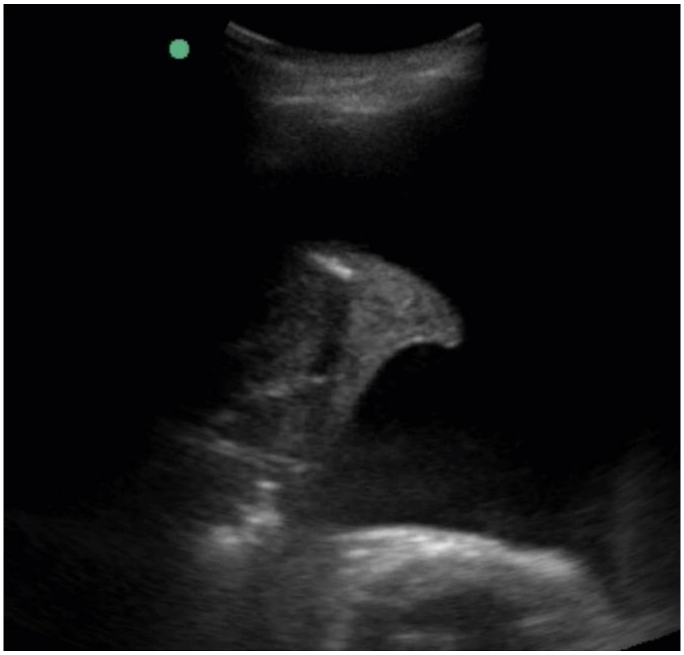

   a. Pleural effusion with atelectasis

b. Pneumonia with parapneumonic effusion

c. Pulmonary embolism

d. Pericardial effusion

3. You are caring for a newly patient in the ICU who is admitted for sepsis due to cellulitis. They received three liters of fluids in the emergency department. You are concerned that they may need to be started on norepinephrine to maintain a MAP of 65. Before you start this medication, you perform the ultrasound below to determine if giving additional fluids might benefit the patient instead. What do you see and how does this change you patient management?

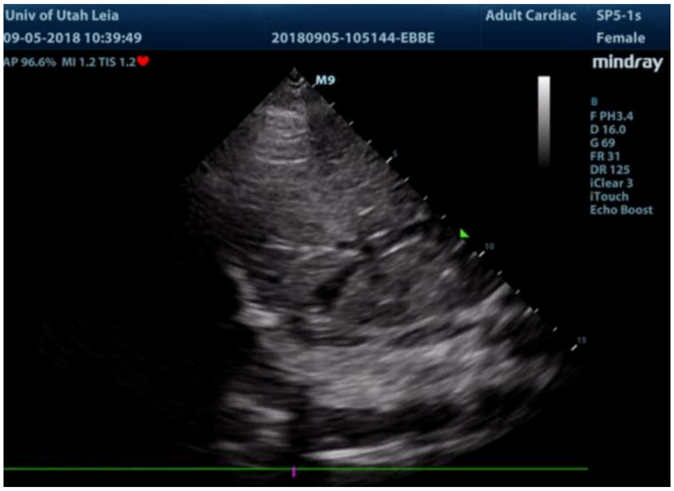

   a. Minimal respiratory variation of the inferior vena cava, so you start norepinephrine.

   b. Minimal respiratory variation of the inferior vena cava, so you give additional IV fluids.

   c. Presence of respiratory variation of the inferior vena cava, so you start norepinephrine.

   d. Presence of respiratory variation of the inferior vena cava, so you give additional fluids.

4. A patient presents to your urgent care due to left sided upper back and lateral chest pain. He recently had a cold and was feeling better. He also reports significant fatigue and continued cough today. He denies fevers, productive cough, or other complaints. You see the image below in the left posterolateral chest only. What do you see? What is the most likely diagnosis?

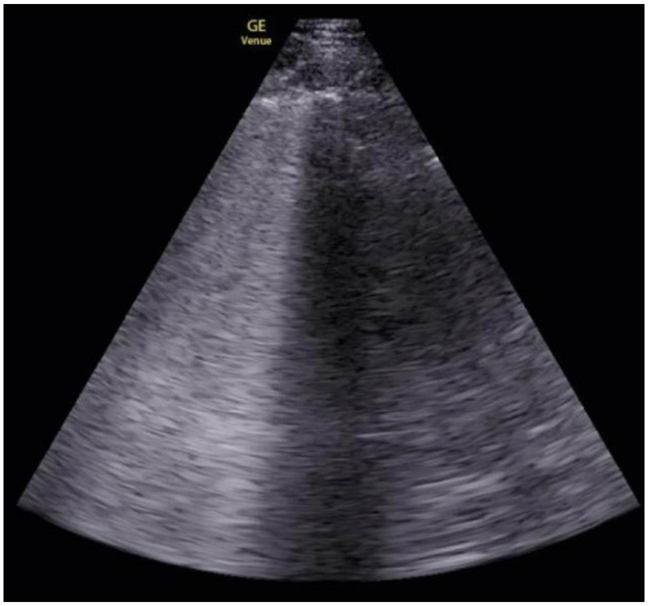

   a. B lines, pulmonary edema

   b. B lines, pneumonia

   c. Consolidation, pneumonia

   d. Consolidation, pulmonary embolism

5. You are seeing your patient with a history of heart failure in clinic. She reports some increased shortness of breath with exertion. Her vitals are normal and she was able to walk in the office without difficulty. She denies any other symptoms and her physical exam is reassuring at this time. Your focused lung ultrasound shows the image below. You increase the patient's diuretic dose and arrange for her to be seen by cardiology in a week for follow up. You also ordered a chest xray and the patient gets a negative result. She is confused about the conflict between the imaging results. What is the mostly likely diagnosis based on the xray and ultrasound findings? Should the patient continue taking the increased dose of diuretic with a negative chest xray?

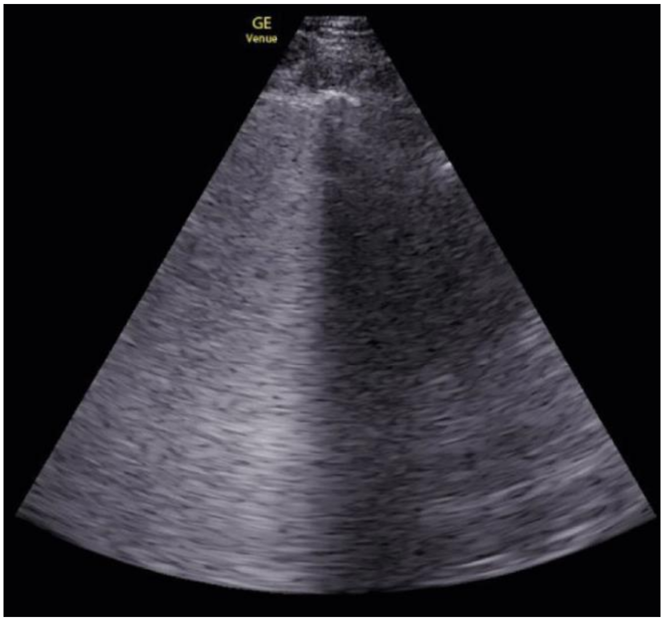

   a. COPD. Yes, she should continue taking her increased diuretic dose because ultrasound and xray findings are consistent with COPD.
   b. COPD. No, should should not continue taking her increased diuretic dose because xray is more senstive than ultrasound for the diagnosis of COPD.
   c. **Pulmonary edema. Yes, she should continue taking her increased diuretic dose because ultrasound is more sensitive than xrays for this diagnosis.**
   d. Pulmonary edema. No, she should not continue taking her increased diuretic dose because the conflict in imaging suggests an alternative diagnosis.

6. You are caring for a patient who recovered from covid-19 1 week ago. He was feeling fine until two days ago. Now he is having chest pain. He does not have shortness of breath, light headedness, fever, or other complaints. What is the diagnosis?

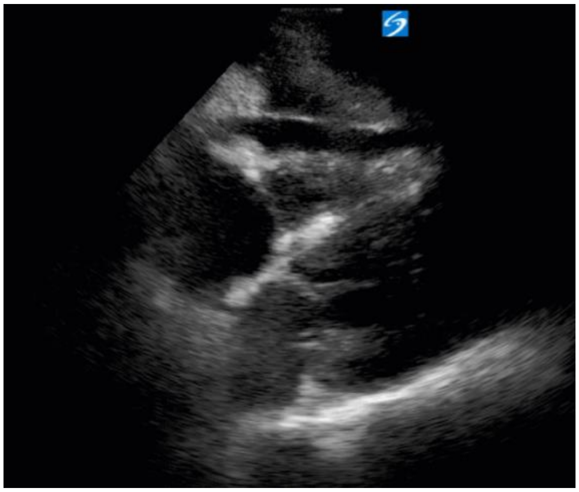

   a. Pleural effusion
   b. **Pericardial effusion**
   c. Ascites
   d. Pneumonia

7. You are establish care for a 50 year old male in a free clinic for the homeless. In take vitals show an oxygen saturation of 88% and a heart rate of 102. He reports exertional fatigue and shortness of breath for several months. He denies chest pain. His physical exam reveals mild wheezes and mild lower extremity edema. He reports a history of untreated COPD. He used meth daily; but denies alcohol, tobacco, or other drugs. His lung ultrasound shows B lines and the image below. What is the likely cause of his symptoms and vitals?

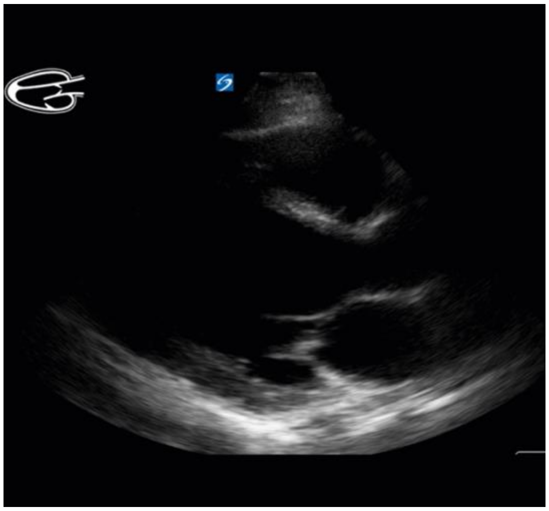

    a. Meth induced myocarditis
    b. COPD flare
    c. **Meth induced heart failure**
    d. Interstitial pulmonary fibrosis

8. You are caring for a hospitalized patient. They have been hospitalized for an acute kidney injury. Their creatine is 2.5. You confirm the patient has a new diagnosis of left ventricle systolic failure during your initial work up. You wonder if this patient should receive fluids to treat their acute kidney injury, so you obtain the IVC clip below. What is your interpretation and how cautious should be about giving this patient fluids?

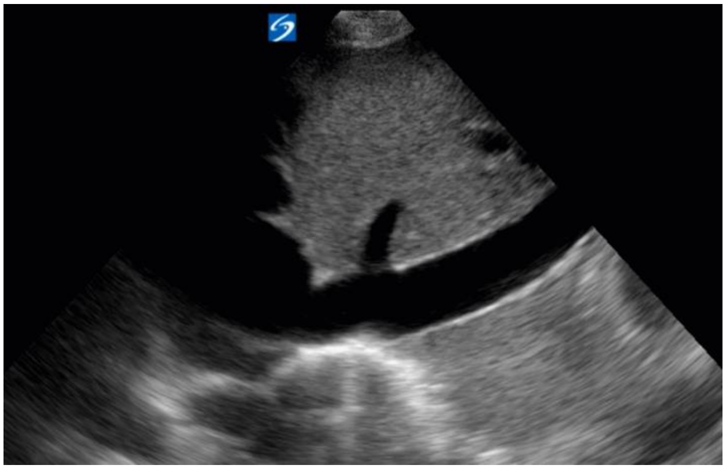

    a. There is sufficient respiratory variation, so you can give IV fluids freely.
    b. **There is minimal to no respiratory variation, so you should consider alternative treatment options to fluids.**
    c. There is 25% respiratory variation, so you should give fluids and reassess the IVC again after.
    d. There is slight respiratory variation, so you can give IV fluids freely.

9. Your 55 year old primary care patient presents to your office for chest pain. The patient has been having chest pain for the last two months. It is not positional or pleuritic. He notices it the most when he is working at his job delivering mail. It improves when he

rests in his mail truck. His EKG, physical exam, and vitals are normal. You obtain the ultrasound image below. What is the next step in treating this patient?

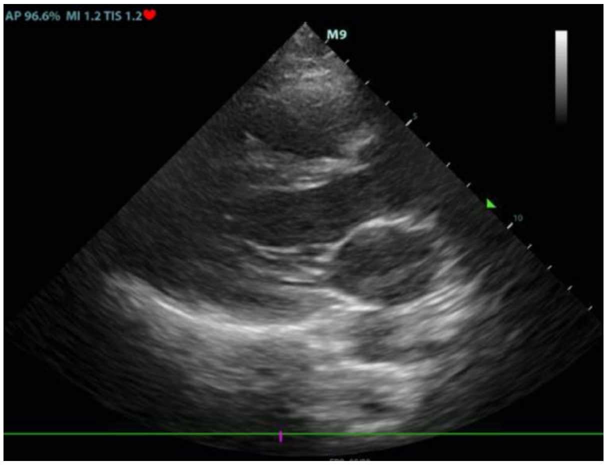

a. The left ventricle has poor squeeze, so you initiate treatment for heart failure.

b. There is pericardial effusion, so you initiate treatment for pericarditis.

c. There is a pleural effusion, so you drain it and analyze the fluid.

d. The is no visible pathology, so you continue working this patient up for other diagnoses.

10. In this parasternal long view of the heart what is the fluid marker by the star?

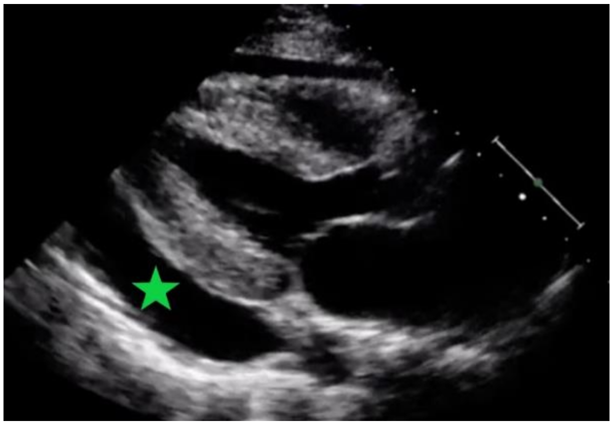

a. Blood in the left ventricle

b. Pleural effusion

c. Pericardial effusion

d. Descending thoracic aorta
