# Supplementary material for: Integrating a self-directed ultrasound curriculum for the internal medicine clerkship
Source: Ultrasound J. 2024 Mar 5;16:19. doi: 10.1186/s13089-024-00367-4 (PMC10914648; doi:10.1186/s13089-024-00367-4)
Supplement: Supplementary file 2 — Supplementary Material 2 [file 13089_2024_367_MOESM2_ESM.pdf]

Supplemental Material B: End of Course Survey

End-of-Course Survey Ultrasound Feedback Questions

Were there any other positive benefits to the ultrasound experience? If so, please comment on those benefits:

Were there any barriers to getting the most out of the ultrasound experience? If so, please provide feedback on what could be changed / improved?

Please rate how much the Ultrasound experience in this clerkship enhanced:

	Not at all	Slightly	Moderately	Extremely
**My learning of pathophysiology / disease processes**				
**My clinical reasoning**				
**My value to the team**				
**Patient Care**				
